# Type 2 diabetes is associated with decreased PGC1α expression in epicardial adipose tissue of patients with coronary artery disease

**DOI:** 10.1186/s12967-016-0999-1

**Published:** 2016-08-19

**Authors:** Inmaculada Moreno-Santos, Luis Miguel Pérez-Belmonte, Manuel Macías-González, María José Mataró, Daniel Castellano, Miguel López-Garrido, Carlos Porras-Martín, Pedro L. Sánchez-Fernández, Juan José Gómez-Doblas, Fernando Cardona, Eduardo de Teresa-Galván, Manuel Jiménez-Navarro

**Affiliations:** 1 Unidad de Gestión Clínica Área del Corazón, Instituto de Investigación Biomédica de Málaga (IBIMA), Hospital Universitario de Málaga (Virgen de la Victoria), Universidad de Málaga, Red de Investigación Cardiovascular (RIC), Málaga, Spain; 2 Unidad de Gestión Clínica de Endocrinología y Nutrición, Instituto de Investigación Biomédica de Málaga (IBIMA), Hospital Universitario de Málaga (Virgen de la Victoria), Universidad de Málaga, CIBER Pathophysiology of Obesity and Nutrition, Málaga, Spain; 3 Servicio de Cardiología, Hospital Universitario de Salamanca, Instituto de Investigación Biomédica de Salamanca (IBISAL), Red de Investigación Cardiovascular (RIC), Universidad de Salamanca (USAL), Salamanca, Spain

**Keywords:** Epicardial adipose tissue, Peroxisome proliferator-activated receptor gamma coactivator 1-alpha (PGC1α), Coronary artery disease, Type 2 diabetes mellitus

## Abstract

**Background:**

Although recent studies indicate that epicardial adipose tissue expresses brown fat-like genes, such as PGC1α, UCP1 and PRDM16, the association of these genes with type 2 diabetes mellitus (DM2) in coronary artery disease (CAD) remains unknown.

**Methods:**

PGC1α, UCP1, and PRDM16 mRNAs expression levels were measured by real-time PCR in epicardial and thoracic subcutaneous adipose tissue from 44 CAD patients (22 with DM2 [CAD-DM2] and 22 without DM2 [CAD-NDM2]) and 23 non-CAD patients (NCAD).

**Results:**

The CAD-DM2 patients had significantly lower PGC1α and UCP1 expression in epicardial adipose tissue than the CAD-NDM2 and NCAD patients. However, PGC1α and UCP1 mRNA trended upward in subcutaneous adipose tissue from CAD-DM2 patients. At multiple regression analysis, age, body mass index, left ventricular ejection fraction, UCP1 expression of epicardial adipose tissue and diabetes came out to be independent predictors of PGC1α levels. Epicardial adipose tissue PGC1α expression was dependent on the number of injured coronary arteries and logistic regression analysis showed that PGC1α expression in epicardial adipose tissue could exert a protective effect against coronary lesions.

**Conclusions:**

DM2 is associated with decreased expression of PGC1α and UCP1 mRNA in epicardial adipose tissue of patients with CAD, likely reflecting a loss of brown-like fat features. Decreased expression of PGC1α in human epicardial adipose tissue is associated with higher prevalence of coronary lesions.

## Background

In contrast to subcutaneous adipose tissue (SAT), epicardial adipose tissue (EAT) represents a visceral fat depot that has not been fully characterized and that has gained significant attention in recent years. EAT is located between the myocardium and the inner layer of visceral pericardium [1]. Thus, due to the close anatomical proximity to the heart and the absence of fascial boundaries, EAT has been implicated in the development of coronary disease artery (CAD).

Several studies have shown that EAT is associated with the development and progression of coronary atherosclerosis, mainly through a dysbalance of pro/anti-inflammatory adipokines production in pathological conditions [2–4]. Indeed, it has been demonstrated that the volume of EAT correlates with the extent and severity of CAD [4–6]. EAT participates in the energy homeostasis of the heart and the vessels [7, 8]; thus the functional EAT might play a protector role over the myocardium or coronary arteries [9]. However, in patients with type 2 diabetes mellitus (DM2), the EAT dysfunction might be the main connection between the diabetic state and complexity of coronary lesions in patients with CAD. DM2 is associated with more extensive CAD, a more aggressive course and greater morbidity and mortality than in coronary patients without DM2 [10].

Brown adipose tissue is a specialized tissue the function of which is to produce heat. Adipose mitochondria play an important role in fatty acid oxidation and adaptive thermogenesis, key adipose-specific metabolic pathways that are regulated by peroxisome proliferator-activated receptor gamma coactivator 1-alpha (PGC1α) [11, 12]. These pathways oxidize lipids and dissipate energy in the form of heat due to the uncoupling of the mitochondrial electron transport chain from the generation of ATP by uncoupling protein 1 (UCP1). On the basis of hundreds of studies, PGC1α is now recognized as a master regulator of mitochondrial biogenesis and oxidative metabolism in many cell types.

Recent studies have reported that human EAT expresses genes of the brown-like adipocytes, such as PGC1α, (UCP1), and PR-domain-missing 16 (PRDM16), suggesting that EAT may play a role in thermogenesis [13, 14]. However, it remains to be determined whether the brown fat-like gene expression in EAT is altered in CAD patients according to diabetes status. Therefore, our main objective was to evaluate the expression of PGC1α, UCP1 and PRDM16 mRNAs in EAT contiguous with CAD in patients with and without DM2. We hypothesized that the gene expression of thermogenic genes in EAT would be altered according to diabetes status, since adipose thermogenesis has been shown to affect excess lipids and fat accumulation [15, 16]. An additional aim was to determine the possible association between brown fat-like gene expression and various biochemical and clinical parameters that it could explain the prevalence of coronary lesions in diabetic patients.

## Methods

### Patients

A total of 44 patients who underwent coronary artery bypass grafting CABG (CAD group, 38 men and six women) and 23 patients who underwent aortic and/or mitral valve replacement (patients without CAD (NCAD), 12 men and 11 women) were enrolled in this study. The CAD group was divided into two groups: those with DM2 (CAD-DM2, 18 men and four women) and those without DM2 (CAD-NDM2, 20 men and two women). The CAD group was defined by the presence of at least one coronary stenosis ≥50 % of luminal diameter by coronary angiogram. The NCAD group (12 men and 11 women) had chronic valvular heart disease with or without stenosis less than 50 % in any vessel requiring valve replacement but not CABG and without DM2. Exclusion criteria were acute inflammatory disease, severe infective disease and/or cancer, and women who were taking hormone replacement therapy. DM2 was defined as having a history of diabetes diagnosed and/or treated with medication, fasting blood glucose ≥126 mg/dl and/or glycated hemoglobin (HbA1c) ≥6.5. All patients gave written informed consent, and the study was reviewed and approved by the Ethics and Research Committee of Virgen de la Victoria Clinical University Hospital, Malaga, Spain, and carried out in accordance with the Declaration of Helsinki.

### Biological material

Human EAT biopsy samples (average 0.2–0.5 g) were taken near the proximal right coronary artery and SAT (average 1.5–2 g) was obtained from the thorax. Thoracic SAT biopsies were taken 30–45 min after anesthesia and EAT biopsies were taken approximately 1 h after anesthesia. All the tissues were frozen immediately in liquid nitrogen and stored at −80 °C for RNA isolation.

### Blood assays

On the morning of surgery, peripheral venous blood was drawn into pyrogen-free tubes with or without EDTA as an anticoagulant. For serum, the tubes were left at room temperature for 20 min and then centrifuged at 1500*g* for 10 min at 4 °C. Fasting glucose, HbA1c, total cholesterol, low-density lipoprotein (LDL), high-density lipoprotein (HDL), triglycerides and creatinine were measured in a Dimension autoanalyzer (Dade Behring Inc., Deerfield, IL) by enzymatic methods (Randox Laboratories, Ldt., UK) in the hospital laboratory.

### RNA isolation and TaqMan real-time reverse transcription–polymerase chain reaction

Adipose tissue samples were minced in TriZol reagent (Invitrogen) and homogenized completely on ice. Total RNA was extracted by chloroform and purified through RNeasy minicolumns. After on-column DNase treatment, RNA was eluted with Rnase-free water. Total RNA was quantified with a spectrophotometer (Nanodrop N-100, Thermo Scientific), and all samples had a 260/280 nm absorbance ratio ≥1.8. Reverse transcriptions were performed using 1 μg of total RNA with Transcriptor First Strand cDNA Synthesis Kit (Roche) and random hexamers in 20 μl reaction. The gene expression levels in the adipose tissue were determined by real time quantitative polymerase chain reaction (PCR) using a predesigned and validated Taqman primer/probe sets [UCP1 (Hs00222453_m1, RefSeq NM_021833.4), PGC1α (Hs01016719_m1, RefSeq NM_013261.3), PRDM16 (Hs00922674_m1, RefSeq NM_022114.3) and cyclophilin A (Hs99999904_m1, RefSeq NM_021130.3)]. Real-time PCR amplifications were performed on 96-well plates in reaction buffer containing Taqman Universal PCR Master Mix (No AmpErase UNG, Applied Biosystems, USA), 150 nM Taqman probe, 900 nM primers, and 22.5 ng cDNA. PCR reaction conditions were 48 °C for 30 min, 95 °C for 10 min, followed by 40 cycles of 95 °C for 15 s and 60 °C for 1 min using an ABI 7500 Fast Detection System (Applied Biosystems). Data were obtained as Ct values according to the manufacturer’s guidelines (the cycle number at which logarithmic PCR plots cross a calculated threshold line) and were used to determine ΔCt values (ΔCt = Ct of the target gene minus Ct of the housekeeping gene). Cyclophilin A transcripts were amplified in the same reaction to normalize for variance in input RNA. mRNA expression levels relative to cyclophilin A were calculated by the 2^−ΔCt^ method. All tests were performed in duplicate. A negative control, RNA amplification without previous retrotranscription, was done to test for possible genomic DNA contamination.

### Statistical analysis

Normality of continuous variables was checked by means of the Kolmogorov–Smirnov test. Continuous variables are expressed as mean ± standard deviation, and as the mean ± SEM in figures and compared by the use of analysis of variance and the post hoc analysis Bonferroni test or with the Student’s test. Differences between EAT and SAT were analyzed with the paired Student’s t test. Categorical variables expressed as proportion and compared by use of Chi squared test. The Pearson correlation coefficient was calculated to estimate the linear correlations between variables. Independent predictors of EAT PGC1α mRNA levels were determined by multiple regression analysis and variables achieving P < 0.05 on correlation analysis were included. To define the risk factors of the development of coronary lesions, multiple logistic regression analysis was used and odds ratios (OR) were calculated. The inclusion of 3 patients in each group, for a a-value of 0.05, (assuming a difference of 1.28 and a standard deviation of 0.28), has the power to detect a significance difference of 95 %. Statistical analyses were performed with SPSS for Windows version 15 (SPSS Inc. Chicago, IL, USA). Values were considered to be statistically significant when P < 0.05.

## Results

### General characteristics of the patients

The general characteristics of the patients in the three groups are summarized in Table 1. No differences between the group of CAD patients with and without DM2 were found for either established risk factors, consumption of medications or general clinical characteristics. Diabetic treatment of CAD patients with DM2 was: only diet (n = 4, 19 %), oral anti-diabetic (n = 12, 57.1 %), oral anti-diabetic and insulin (n = 4, 19 %) and only insulin (n = 1, 4.9 %). No patient was taking thiazolidinediones. Percentage of CAD-DM2 patients on oral-antidiabetic were: metformin, 73 %, sulfonylureas (gliclazide and glibenclamide), 23 %.

**Table 1:** Clinical and laboratory characteristics of coronary patients according to the presence of type 2 diabetes mellitus, and non-CAD patients

	NCAD	CAD-NDM2	P value*	CAD-DM2	P value**	P value***	P value
Gender, n (M/F)	12/11	20/2	0.007	18/4	0.057	0.664	0.01
Age (years)	62 ± 11	63 ± 11	0.762	65 ± 9	0.323	0.513	0.619
BMI (kg/m^2^)	27.8 ± 5.3	28.2 ± 6.4	0.820	29.8 ± 5.7	0.229	0.386	0.481
LVEF (%)	57.7 ± 12.4	51.7 ± 8.0	0.05	51.2 ± 4.2	0.024	0.796	0.031
Risk factors, n (%)							
Dyslipidemia	10 (43.5)	15 (68.2)	0.136	18 (81.8)	0.013	0.487	0.021
Hypertension	21 (91.3)	17 (77.2)	0.242	20 (90.9)	0.99	0.412	0.296
Current smoking	7 (30.4)	14 (63.6)	0.04	7 (31.8)	0.99	0.07	0.04
CVA	2 (8.7)	1 (4.5)	0.578	2 (9.1)	0.962	0.549	0.812
Medications, n (%)							
Aspirin	13 (56.5)	8 (36.4)	0.236	14 (63.6)	0.763	0.131	0.170
Statin	9 (39.1)	8 (36.4)	0.85	12 (54.5)	0.376	0.364	0.421
ACEI/ARB	6 (26.1)	5 (22.7)	0.796	10 (45.4)	0.221	0.202	0.213
Beta-blocker	15 (65.2)	8 (36.4)	0.076	14 (63.6)	0.912	0.131	0.094
Biochemical data							
Glucose (mg/dL)	106.00 ± 18.93	103.00 ± 20.10	0.61	153.86 ± 41.51	<0.0001	<0.0001	<0.0001
Cholesterol (mg/dL)	161.88 ± 41.98	165.47 ± 30.17	0.74	143.88 ± 34.29	0.12	0.032	0.109
LDL-cholesterol (mg/dL)	97.24 ± 31.76	102.95 ± 27.1	0.52	81.68 ± 23.29	0.068	0.008	0.04
HDL-cholesterol (mg/dL)	40.70 ± 14.32	36.47 ± 11.43	0.28	29.75 ± 6.44	0.002	0.020	0.008
Triglycerides (mg/dL)	119.76 ± 44.59	153.23 ± 61.3	0.041	187.58 ± 55.12	<0.0001	0.057	0.0005
HbA1c	5.46 ± 0.63	5.91 ± 0.61	0.02	7.51 ± 0.90	<0.0001	<0.0001	<0.0001
Creatinine (mg/dL)	1.00 ± 0.32	1.24 ± 0.80	0.16	1.20 ± 0.51	0.11	0.79	0.329

Comparison between the results of the different groups was made with the Student’s t test or with the analysis of variance (ANOVA) and Chi square test for continuous and categorical data, respectively. Values were considered to be statistically significant when P < 0.05

*CAD* coronary artery disease; *n* no. of subjects; *BMI* body mass index; *LVEF* left ventricle ejection fraction; *CVA* cerebrovascular accident; *ACEI* angiotensin converting enzyme inhibitor; *ARB* angiotensin II receptor blocker; *LDL* low-density lipoprotein; *HDL* high-density lipoprotein; *HbA1c* glycated hemoglobin

p value: overall comparison for all groups

p value*: NCAD vs CAD-NDM2 comparison

p value**: NCAD- vs CAD-DM2 comparison

p value***: CAD-NDM2 vs CAD-DM2 comparison

### Thermogenic gene mRNA expression in EAT and SAT

**Fig. 1:**
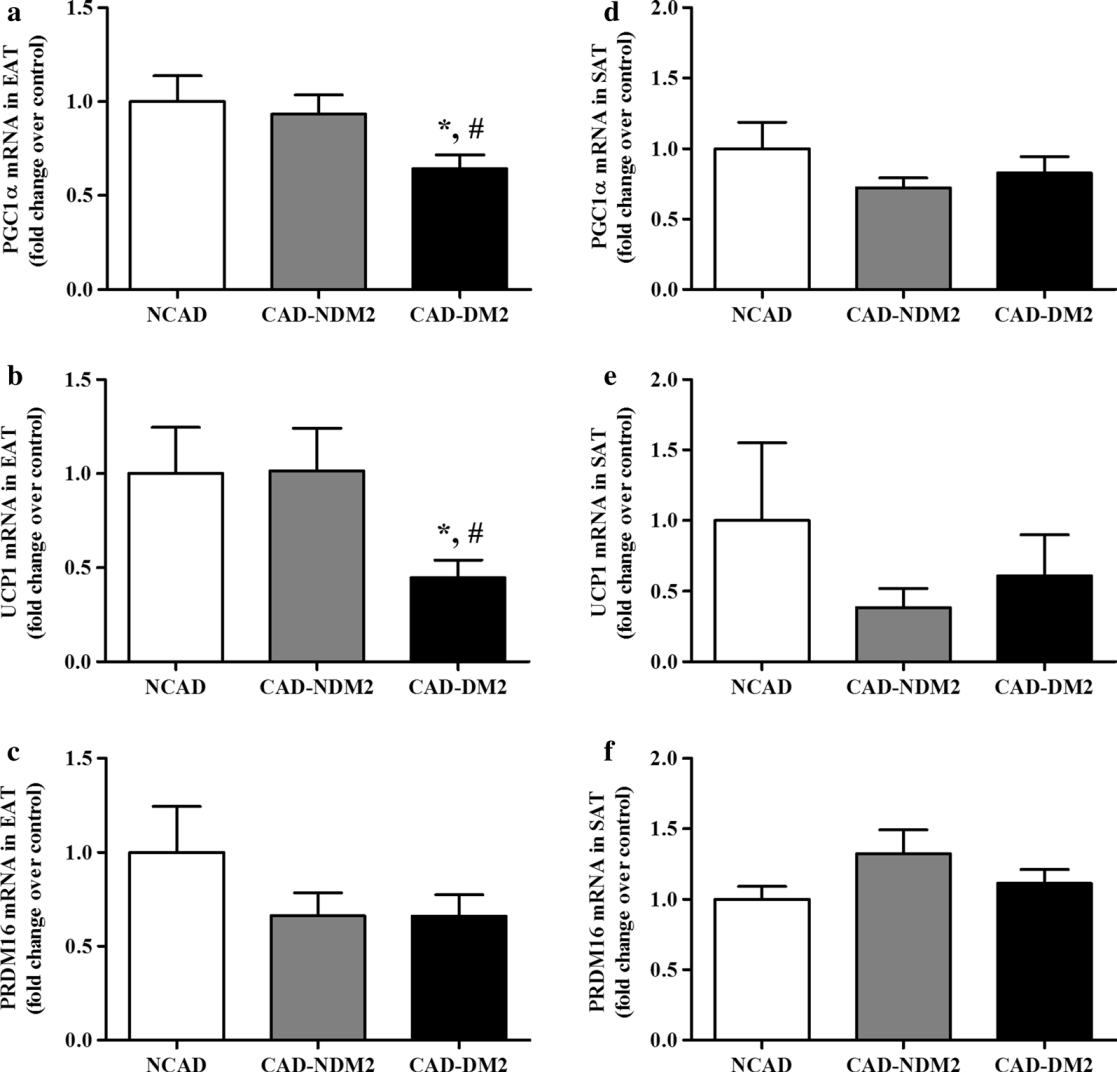
PGC1α, UCP1 and PRDM16 mRNA expression levels in EAT and SAT. TaqMan^*®*^ real time PCR for PGC1α, UCP1 and PRDM16 was performed on human EAT (**a**–**c**) and thoracic SAT (**d**–**f**) from coronary artery disease (CAD) patients with type 2 diabetes (CAD-DM2) (n = 22) and without it (CAD-NDM2) (n = 22) and non-CAD patients (NCAD) (n = 23). Results were expressed as the mean ± SEM of the completed experiment in duplicate. *P < 0.05 vs CAD-NDM2 and ^#^P < 0.05 vs NCAD. *PGC1α* peroxisome proliferator-activated receptor gamma coactivator-1 alpha; *UCP1* uncoupling protein 1; *PRDM16* PR domain containing 16; *EAT* epicardial adipose tissue; *SAT* subcutaneous adipose tissue

To investigate possible differential expression between the two groups of CAD patients (DM2 and NDM2) and NCAD, mRNA expression levels of EAT and SAT brown fat genes were evaluated. Values are gives as fold increase relative to the NCAD group and differences between groups were considered significant at P < 0.05 (Fig. 1).

PGC1α and UCP1 mRNAs in EAT were significantly lower in the CAD-DM2 patients compared with the CAD-NDM2 patients (P = 0.027 and P = 0.038, respectively) and NCAD patients (p = 0.031 and p = 0.045, respectively) (Fig. 1a, b). However, in contrast to EAT, there was a trend for higher PGC1α and UCP1 mRNA expression levels in the SAT from the CAD-DM2 patients compared with the CAD-NDM2 patients, though it was not statistically significant (Fig. 1d, e). PRDM16 mRNA expression was lower in the EAT (Fig. 1c) and higher in the SAT (Fig. 1f) from the CAD group compared with NCAD, but it was not statistically significant.

### Comparison of the mRNA level of thermogenic genes from epicardial versus thoracic subcutaneous fat tissue in patients with CAD

**Fig. 2:**
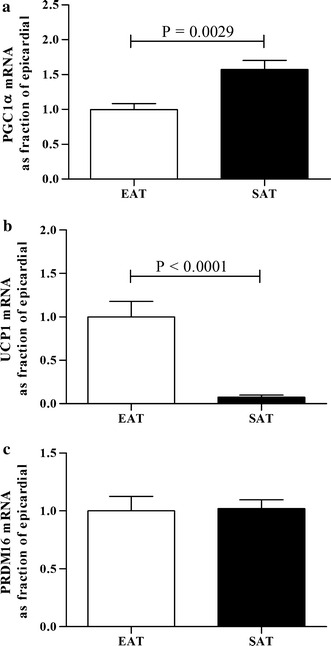
Comparison of PGC1α, UCP1 and PRDM16 mRNAs in EAT and SAT. TaqMan^®^ real-time PCR analysis for mRNA expressions of PGC1α (**a**), UCP1 (**b**) and PRDM16 (**c**) in human adipose tissues (EAT and SAT) from coronary artery disease (CAD) patients. mRNA expression in thoracic SAT were compared as a fraction of epicardial fat, which was arbitrarily assigned the value of 1. Results were expressed as the mean ± SEM of the completed experiment in duplicate (n = 36). *PGC1α* peroxisome proliferator-activated receptor gamma coactivator-1 alpha; *UCP1* uncoupling protein 1; *PRDM16* PR domain containing 16; *EAT* epicardial adipose tissue; *SAT* subcutaneous adipose tissue

To elucidate potential pathophysiological implications in gene expression, we compared the expression profile of the target genes involved in thermogenic and mitochondrial function in the EAT and SAT of patients with CAD (Fig. 2).

PGC1α mRNA levels were significantly higher (1.6-fold) in thoracic SAT that in EAT (P = 0.0029). Conversely, UCP1 mRNA levels were significantly lower (13-fold) in thoracic SAT than in EAT (P < 0.0001). PRDM16 mRNA was similar in EAT and SAT.

### Association between PGC1α mRNA expression in epicardial and thoracic subcutaneous adipose tissue and clinical variables

**Fig. 3:**
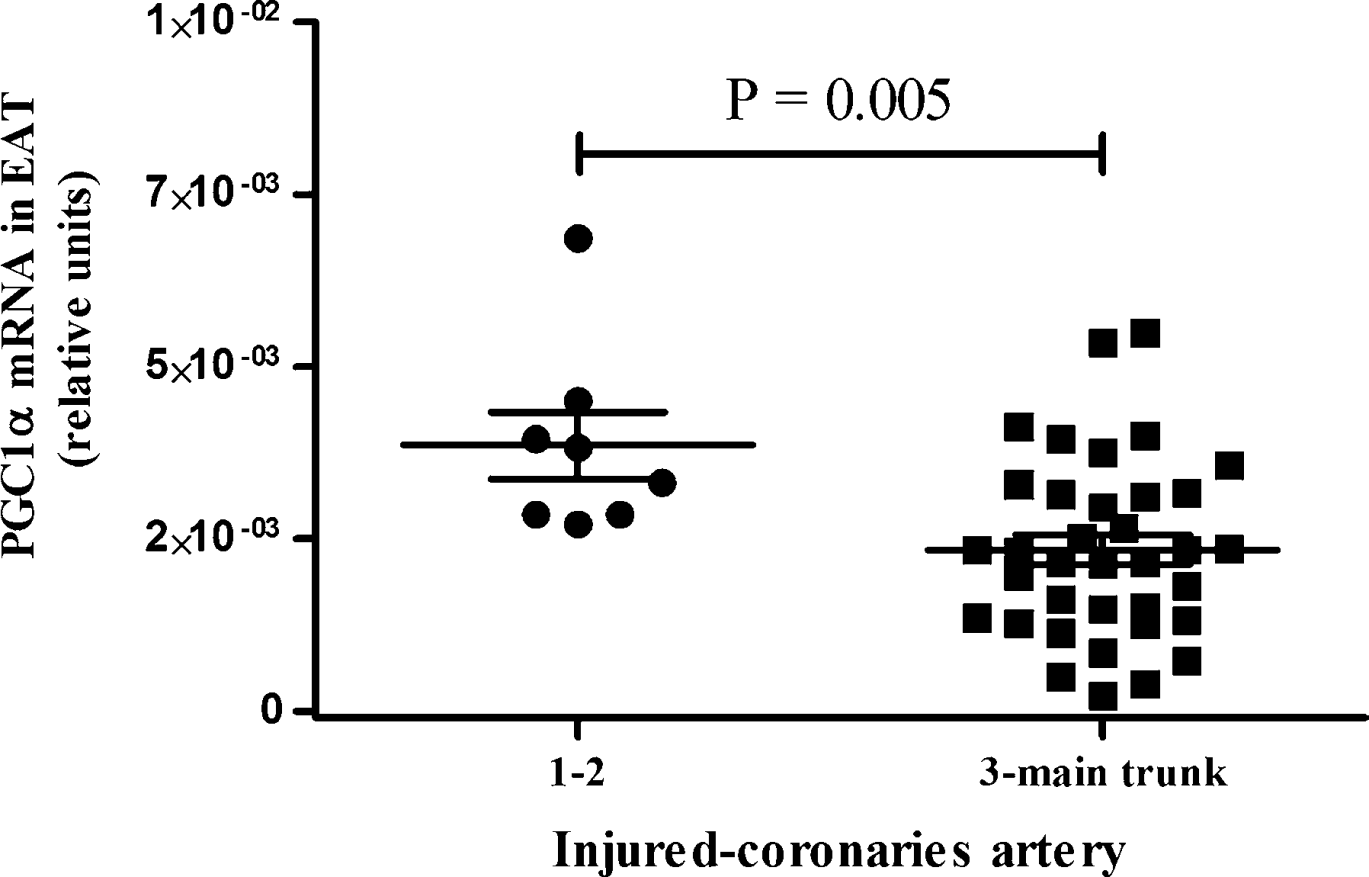
Association between PGC1α mRNA expression in EAT and coronary lesions. *Dot plots* represent PGC1α mRNA levels detected by real-time PCR in the group of patients regarding injured coronary arteries (1 or 2 and 3 or main trunk). Student’s t test was used to determine differences among the groups. P = 0.005. *PGC1α* peroxisome proliferator-activated receptor gamma coactivator-1 alpha; *EAT* epicardial adipose tissue, *N* number of subjets

The PGC1α mRNA expression levels in EAT correlated positively with HDL cholesterol (r = 0.498, P = 0.004), LVEF (r = 0.376, P = 0.014), and EAT UCP1 mRNA expression (r = 0.307, P = 0.043) and negatively with the BMI (r = −0.431, P = 0.025), serum levels of triglycerides (r = −0.468, P = 0.005). Indeed, EAT PGC1α mRNA expression levels differed with the number of injured coronary arteries (P = 0.0005) (Fig. 3). Thus, PGC1α mRNA expression was higher in patients with one or two injured coronary arteries (2^−ΔCt^ = 0.0038 ± 0.0014) than in those with three or left main trunk injured coronary arteries (2^−ΔCt^ = 0.0023 ± 0.0013). Considering only men, EAT PGC1α mRNA expression levels with the number of injured vessels remained statistically significant (P = 0.020). Multiple regression analysis showed that BMI (β = −0.081, P = 0.048; 95 % CI −0.162, −0.001), LVEF (β = 0.043, P = 0.006, 95 % CI 0.013, 0.072), age (β = 0.072, P = 0.003, 95 % CI 0.026, 0.117), EAT UCP1 mRNA levels (β = 0.148, P = 0.001, 95 % CI 0.063, 0.233) and DM2 (β = −0.929, P = 0.044, 95 % CI −1.832, −0.026) were independently associated with EAT PGC1α mRNA levels (Table 2). Finally, logistic regression analysis showed the PGC1α mRNA expression in EAT to be a protective factor against the number of injured coronary arteries (OR = 0.310; P = 0.037, 95 % CI = 0.103, 0.931). However, circulating triglyceride levels were associated with a higher risk of suffering coronary lesions (OR = 1.023; P = 0.044, 95 % CI = 1.001, 1.046; p < 0.05) (Table 3).

**Table 2:** Multiple regression analysis for prediction of EAT PGC1α mRNA levels

Variable	EAT PGC1α mRNA (R = 0.789; R^2^ = 0.623)			
	β	95 % CI lower	95 % CI upper	P value
Age	0.072	0.026	0.117	0.003
BMI	−0.081	−0.162	−0.001	0.048
Gender	−0.848	−1.841	0.146	0.092
DM2	−0.929	−1.832	−0.026	0.044
LVEF	0.043	0.013	0.072	0.006
Dyslipidemia	0.272	−0.578	1.122	0.518
Triglycerides	0.007	0.000	0.014	0.053
EAT UCP1 mRNA	0.148	0.063	0.233	0.001

Values were considered to be statistically significant when P < 0.05

*EAT* epicardial adipose tissue; *PGC1α* peroxisome proliferator-activated receptor gamma coactivator 1 alpha; *CI* confidence interval; *BMI* body mass index; *DM2* type 2 diabetes mellitus; *LVEF* left ventricle ejection fraction; *UCP1* uncoupling protein 1

**Table 3:** Logistic regression analysis for prevalence of coronary lesions

Variable	OR (95 % CI)	P value
PGC1α mRNA	0.310 (0.103–0.931)	0.037
Age	1.137 (0.954–1.355)	0.151
Gender (man)	27.908 (0.823–946.906)	0.064
BMI	0.948 (0.774–1.162)	0.608
Dyslipidemia	3.240 (0.332–31.602)	0.312
Triglycerides	1.023 (1.001–1.046)	0.044

Values were considered to be statistically significant when P < 0.05

*OR* odds ratio; *CI* confidence interval; *PGC1α* peroxisome proliferator-activated receptor gamma coactivator 1 alpha; *BMI* body mass index

## Discussion

Although there is existing research linking brown fat-like gene expression in EAT to the metabolic syndrome and DM2 [13], this is the first study to focus on EAT and SAT brown fat-like gene expression from CAD patients with DM2 itself.

Our main finding was that CAD patients with DM2 expressed significantly lower PGC1α and UCP1 mRNA levels in EAT than those without DM2 and NCAD patients. Conversely, the mRNA expression of these brown fat-like genes trended upward in SAT from CAD patients with DM2 and NCAD patients. We found that EAT PGC1α mRNA levels correlated positively with HDL-cholesterol, LVEF, and EAT UCP1 mRNA levels and negatively with BMI, circulating triglycerides. Multiple regression analysis showed that age, BMI, LVEF, EAT UCP1 expression and diabetes were independent predictors of PGC1α mRNA levels in EAT. We also found that PGC1α expression levels in EAT decreased with the number of injured coronary arteries and EAT PGC1α was shown as a possible protective factor against coronary lesions.

In line with previous research [14, 17], we found that UCP1, the specific marker of brown adipocytes, is expressed in human EAT, confirming the possibility that EAT functions like brown adipose tissue. It has been reported that beta adrenergic stimulation cannot induce UCP1 in brown adipose tissue that lacks PGC1α activity [16]. Interestingly, most factors that induce or repress the browning program act through modulation of PGC1α activity [12]. Therefore, the lower expression of EAT PGC1α and UCP1 from CAD patients found in our study suggests a loss of EAT brown-like features in DM2, which may act detrimentally on metabolism [18, 19] and promote the progression and severity of CAD [9].

To determine possible regional differences of fat tissue and brown fat-like gene expression, fat depots of epicardial and thoracic adipose tissues were compared in each CAD patient. We found that EAT exhibited significantly higher expression of UCP1 and lower expression of PGC1α than thoracic SAT. Our data confirm and extend previous results demonstrating higher UCP1 mRNA expression in EAT than in SAT [13, 17]. However, the significantly reduced PGC1α mRNA expression in EAT found in our study is in contrast with the earlier studies, which found that PGC1α mRNA expression was higher in EAT [13, 17]. This might be due to a different number of diabetic patients in each study, as we showed that PGC1α mRNA expression was altered in each fat depot according to the diabetes status, so it was lower in EAT from diabetic patients, but higher in SAT. Although PGC1α mRNA expression was higher in SAT, its contribution to CAD should be higher in EAT due to its close proximity to the coronary arteries [1]. Thus, we also found a negative association between PGC1α expression in EAT and injured coronary artery number, but SAT PGC1A expression was not associated with greater prevalence of CAD.

PGC1α is pivotal in lipid metabolism and mitochondrial function because it controls genes involved in fatty acid oxidation and the mitochondrial respiratory chain [16, 20]. A reduced expression of genes involved in fatty acid oxidation could be associated with altered lipid uptake in the adipose tissue [7, 21]. Then, a reduced clearance of circulating lipids by adipose tissue would also be expected. Accordingly, circulating triglyceride levels were higher in CAD patients with DM2. Moreover, EAT PGC1α mRNA levels correlated positively with HDL-cholesterol levels and negatively with circulating triglyceride levels. Our data support the involvement of EAT in lipid metabolism proposed by Chechi et al. [17] and the possible contribution of brown adipose tissue to triglyceride clearance and metabolism [18, 22]. The positive correlation found in our study between PGC1α and UCP1 mRNAs in EAT may reflect the regulation of UCP1 expression by PGC1α [11].

The lower PGC1α mRNA expression in EAT from CAD patients with DM2 might contribute to the lower clearance of circulating triglycerides, possibly due to reduced PGC1α and UCP1-mediated β-oxidation of fatty acids in EAT, which may be accompanied by a suppression of adipocyte fatty acid uptake [21, 22]. PGC1α-mediated reduced β-oxidation of fatty acids and EAT-mediated reduced triglyceride clearance might expose the heart to excessively high circulating levels of lipids, leading to lipid-induced cardiotoxicity and, with EAT-mediated lower HDL-cholesterol, which has anti-inflammatory and anti-atherosclerotic properties, increase the risk and the severity of CAD. Moreover, due to EAT and myocardium sharing the same blood supply, a PGC1α and UCP1-mediated reduced β-oxidation of fatty acids in EAT might also alter the functionality of the myocardium such that it does not provide sufficient energy. Thus, we also found that EAT PGC1α mRNA expression correlated positively with LVEF, it was inversely associated with the number of injured coronary arteries and was shown as a protective factor against CAD progression. Together, these findings are also consistent with studies implicating a relationship between adipose mitochondria dysfunction and metabolic and cardiac complications [15, 20, 23].

Numerous studies have investigated the association between PGC1α gene expression in skeletal muscle and DM2, with conflicting results [24]. However, the association of PGC1α mRNA in adipose tissue with and without DM2 and CAD has been less studied, ours being the first study in the epicardial depot. A central question is whether reduced PGC1α and UCP1 mRNA expression in EAT is a prelude to the development of DM2 through a constitutively lower rate of β-oxidation and lower adipose activation, or a consequence of the diabetes itself, in either case leading to increased severity of CAD. Future studies should focus on elucidating mechanisms by which EAT is involved in development and progression of CAD in various disease populations.

### Study limitations

All the subjects were patients with valvular heart disease or CAD. Only small samples of EAT were collected from each patient and they were insufficient for a thorough analysis and correlation between mRNA and protein.

## Conclusions

In conclusion, the results of the study showed that DM2 is associated with decreased PGC1α and UCP1 mRNA expression in EAT of patients with CAD, likely reflecting a loss of brown-like fat features. Another important finding was that a decreased expression of PGC1α in human EAT is associated with higher prevalence of coronary lesions. In addition, PGC1α was shown as possible protective factor against coronary lesions. These low levels of PGC1α, a master regulator of oxidative metabolism and mitochondrial function, could have important repercussions on the pathways regulated by PGC1α in EAT. Thus, PGC1α might represent a possible pharmacological target in diabetes-related coronary disease.

## Abbreviations

ACEI: angiotensin converting enzyme inhibitor
ARB: angiotensin II receptor blocker
BMI: body mass index
CABG: coronary artery bypass grafting
CAD: coronary artery disease
CVA: cerebrovascular accident
DM2: type 2 diabetes mellitus
EAT: epicardial adipose tissue
HDL: high-density lipoprotein
Hb1Ac: glycated hemoglobin
LDL: low-density lipoprotein
LVEF: left ventricular ejection fraction
PGC1α: peroxisome proliferator-activated receptor gamma coactivator 1-alpha
PRDM16: PR-domain-missing 16
SAT: subcutaneous adipose tissue
UCP1: uncoupling protein 1
